# The Effects of Online Behavioral Parenting Interventions on Child Outcomes, Parenting Ability and Parent Outcomes: A Systematic Review and Meta-analysis

**DOI:** 10.1007/s10567-024-00477-4

**Published:** 2024-04-13

**Authors:** John McAloon, Simone Mastrillo Armstrong

**Affiliations:** UTS: Family Child Behavior Clinic, Discipline of Clinical Psychology, Graduate School of Health, University of Technology Sydney, Level 5 Building 20, 100 Broadway, Ultimo, NSW 2007 Australia

**Keywords:** Online, Parenting, Emotion regulation, Child behavior, Meta-analysis, Relationships

## Abstract

The twenty-first century has seen the development and delivery of online programs of behavioral family intervention for disruptive child behavior. Typically, programs evaluate outcomes in terms of change in child functioning and change in parenting ability. Existing research has also articulated the importance of parent–child relational capacity and its role in facilitating change in child functioning, and the importance of parent emotion regulation in the interests of ensuring optimal child development. These factors were explored in a meta-analysis of *k* = 14 prospective longitudinal research studies of online parenting interventions for disruptive child behavior. Peer reviewed randomized controlled trials with inactive control groups that were published in English between 2000 and 2022 were included in the review if they were delivered online; offered parent self-directed treatment; included as participants families who were screened as having child behavioral difficulties on validated psychometric assessment measures; and assessed child treatment outcomes, parenting ability and parent treatment outcomes. The protocol for this study was pre-registered with PROSPERO (CRD42020215947). Statistical analyses employed random effects models and reported pooled effect sizes (Hedge’s *g*) within and between groups. Results emphasize the importance of child outcomes and parenting ability in program assessment, however, suggest that parents’ capacity to develop optimal parent–child relationships and regulate emotion may not be sufficiently reflected in program content. Identified continuous and categorical moderators of treatment outcome were also assessed. Results of the review are discussed in terms of their potential to influence the future development of online programs of behavioral family intervention and, therefore, child development.

## Introduction

The twenty-first century has seen online programs of behavioral family intervention for disruptive child behavior delivered online. The term *behavioral family intervention* accounts for a cluster of treatment components commonly delivered to parent(s) or carer(s) developed on the basis of social learning theory (Bandura, 1977), and operant behavioral principles (Skinner, 1963), and utilised in parent training and behaviorally based family therapy (Blechman, 1981; Sanders & Dadds, 1993). A decade ago, Nieuwboer and colleagues published the first meta-analysis of online treatments for child behavioral problems (Nieuwboer et al., 2013a, 2013b). Nieuwboer’s review was instrumental in establishing the importance of online programs of behavioral family intervention and, since then, numerous systematic reviews and meta-analyses have demonstrated the effectiveness of such programs, particularly in child and parenting outcome terms (Opie et al., 2023; Spencer et al., 2020). The online delivery of programs of behavioral family intervention is now sufficiently well established in the empirical literature that it can be regarded as a mode of treatment delivery in its own right. This review extends current knowledge by reconsidering the basis upon which online programs of behavioral family intervention are developed and identifying areas potential future importance.

The online delivery of behaviorally based family interventions for disruptive child behavior has been a focus of recent treatment research (Leijten et al., 2019; Spencer et al., 2020). Several meta-analyses have assessed outcomes from online programs of behavioral family intervention for disruptive child behavior (Nieuwboer et al., 2013a, 2013b; Spencer et al., 2020). Outcomes from such programs, and the metrics used to assess them, can be considered in three distinct ways. First, and most commonly, research considers the extent to which online programs of behavioral family intervention are able to demonstrate improvements in child behavior (Baumel et al., 2016; Thongseiratch et al., 2020). Secondly, research considers the extent to which changes in parenting ability can be demonstrated to result from online programs of behavioral family intervention (Nieuwboer et al., 2013a, 2013b; Spencer et al., 2020). This can occur in two distinct but mutually beneficial ways. First, parenting ability may be conceptualized as the effectiveness with which parents feel they are able to parent (Baumel et al., 2016; Florean et al., 2020; Thongseiratch et al., 2020). Second, parenting ability may be considered in terms of the extent to which parents are able to develop relationships with their children that foster optimal developmental outcomes (Opie et al., 2023). A third way for research to consider the effectiveness of online programs of behavioral family intervention is by assessing change in parent functioning, as distinct from changes in parenting ability (Spencer et al., 2020; Thongseiratch et al., 2020). This may, for instance, result from improving parents’ regulation of their own emotion, or control of their own behavior. As such, beneficial change in parent stress or parent anger may represent appropriate outcomes from online programs of behavioral family intervention (Thongseiratch et al., 2020).

## Child Outcomes

Numerous studies have documented the effectiveness of online programs of behavioral family intervention. One of the first meta-analytic evaluations drew on *k* = 19 studies and reported on 15 child outcome variables (Nieuwboer et al., 2013a, 2013b). Studies in this review included a range of child and adolescent participants and reported on behaviors and attitudes ranging from child behavioral outcomes to attitudinal change about drug use. Fixed effects models revealed a moderate effect size across child outcomes (Hedges* g* = 0.42) and the authors acknowledged methodological weaknesses in the studies included in the review. Child and adolescent participants were also reviewed in a meta-analysis of *k* = 28 studies that reported on 15 outcome variables (Spencer et al., 2020). Moderate effect sizes were reported in reducing behavioral and anxiety problems across participants (*d* = − 0.58, *d* = − 0.31 respectively). The importance of differentiating between outcomes for children and adolescents was clearly demonstrated in a meta-analysis of *k* = 7 heterogeneous randomized trials comparing the effectiveness of digital programs of behavioral family intervention with waitlist or no treatment controls (Baumel et al., 2016). Significantly greater improvements in the behavior of children aged 3.9–6.8 years with clinically significant symptoms were reported than they were for children aged 11.8–14 years without clinically significant symptoms (*d* = 0.61, *d* = 0.21 respectively).

More recently, meta-analyses of randomized trials of online programs of behavioral family intervention have contributed further to an understanding of both their limitations and potential in improving child behavior. A meta-analysis of *k* = 15 trials demonstrated consistency with previous literature in reporting a moderate effect size (Hedges* g* = 0.40) in reducing child behavior problems regardless of whether the mode of delivery was online or face-to-face (Florean et al., 2020). Combinations of program components were assessed in a second review comprising *k* = 12 studies involving children aged 2–12 years (Thongseiratch et al., 2020). Results confirmed small effects of online programs of behavioral family intervention in reducing child behavioral and emotional problems when compared with waitlist or online control resources (Hedges *g* =  − 0.32, Hedges *g* =  − 0.22 respectively). The only moderator of program success identified in this review was the provision of program participation reminders to parents.

## Parenting Ability

In addition to reporting on child outcomes, it is also important to consider factors that facilitate those outcomes (Shaffer & Obradović, 2017). A recent network meta-analysis identified the importance of parents understanding of, and an ability to use, operant principles and associated schedules of reinforcement (Kjøbli et al., 2023). In contemporary parenting programs, skills in these areas develop initially in response to the recognition and contingent reinforcement of desirable child behavior. Subsequently, it indicates that parents have succeeded in limiting engagement (inadvertent contingent reinforcement) of undesirable child behavior. This capacity, originally articulated by Hanf (1969), has consistently been identified as central to the prevention of behavioral difficulties in children, and is applicable to internalizing as well as externalizing child behavior (McAloon & Lazarou, 2019; Webster-Stratton, 1990).

Parenting ability can also be assessed through parent self-report of parenting effectiveness, otherwise known as parenting self-efficacy. Parenting self-efficacy refers to beliefs parents have about their ability to use their knowledge and skills effectively to carry out parenting related tasks (Bandura, 1977; Coleman & Karraker, 1998). Perhaps unsurprisingly, lower levels of parenting self-efficacy have been associated with problematic parenting practices (e.g., hostility, harsh discipline, and coercion) in both low and high-risk child populations (Bor & Sanders, 2004; Chau & Giallo, 2015). Less self-efficacious parents may resort to problematic parenting practices to manage their child’s behavior which may, in turn, result in greater emotional and behavioral difficulties later in childhood (McAloon & Lazarou, 2019; Rominov et al., 2016).

Perhaps the single most important environmental influence on child development is relational (Fairchild et al., 2019; Opie et al., 2023). The qualities inherent in those relationships have implications for children’s development (Castro et al., 2015; Harold & Sellers, 2018; Morris et al., 2007). Behavioral family systems models (Sein et al., 1987) suggest that the relation between child functioning and parent functioning is transactional such that the qualities of parenting are both determinants of, and result from, child behavioral characteristics (Belsky, 2005; Serbin et al., 2015). Transactional processes influence individual development and adaptation (Crittenden, 2006; Kochanska et al., 2019; Newton et al., 2014) as well as intra-familial relationships (Scott et al., 2018). Gains derived from engagement in online programs of behavioral family intervention cannot occur outside relationships, and the richer those relationships, the greater the likelihood they will facilitate the gains sought (Kaehler et al., 2016). This supports Hanf’s (1969) assertion that parenting programs that deliver behavior management skills and relationally enhancing skills will likely be effective in addressing disruptive child behavior.

## Parent Outcomes

From their earliest relationships, children both experience and develop emotion regulation through direct observation of, and engagement by, their carers (Feldman, 2016; Morris et al., 2017). Toddlers who come from calm, regulated environments do better socially, emotionally, and behaviorally than toddlers who come from dysregulated environments (Crespo et al., 2017; Zimmer-Gembeck et al., 2017). Processes of reciprocal engagement between children and their parents establish a range of secondary environmental influences on the development of emotion regulation (Havighurst et al., 2010). These revolve primarily around the capacity for the expression and regulation of emotion within the behavioral family system, and the extent to which it influences/is influenced by relational transactions within that system (Hajal & Paley, 2020; Morris et al., 2017).

Parental stress has been the subject of much research in the last three decades. Research indicates a relation between parent stress and child behavior, both with respect to child internalising and externalising behavior (Webster-Stratton, 1990). Research further suggests that this relation is transactional in that parent stress both affects, and is affected by, child behavior (Mackler et al., 2015; Webster-Stratton, 1990). Two significant targets of intervention can be identified in parental emotion regulation: these are parent ability to manage internal processes of stress response, and parental ability to moderate the external expression of those responses, commonly in the form of anger. In terms of direct learning, emotion regulatory processes observable to a child may be both internal (for instance, learning about parental cognitive processes involved in the management of stress) and external (for instance, understanding the preferences a parent might display in their behavioral expression of those cognitive processes).

## The Present Review

Parents’ ability to nurture change in their children’s behavior is associated with their own ability to develop relationships capable of facilitating that change (Hajal & Paley, 2020; Shaffer & Obradović, 2017). The primary aim of this review was to assess the potential for online programs of behavioral family intervention to return benefit in three domains of importance. First, child treatment outcomes were assessed as the potential for online programs to demonstrate change in externalising child behavior. Second, parenting ability was assessed as the potential for online programs to facilitate change in parenting self-efficacy and in the quality of parent–child relationships. Finally, parent outcomes were assessed as change in parents’ capacity to regulate emotion in the form of stress, and to control behavior in the form of anger, following the delivery of online programs of behavioral family intervention. Together, these three considerations are important to the extent to which parents and carers can maintain regulated (Castro et al., 2015; Morris et al., 2017; Zimmer-Gembeck et al., 2017), responsive (Blair et al., 2015; Scott et al., 2018) and relationally rich (Kochanska et al., 2019) developmental environments for their children.

## Method

### Protocol and Registration

This review was conducted in accordance with the Preferred Reporting Items for Systematic Reviews and Meta-Analyses (PRISMA) guidelines (Page et al., 2021) and incorporated the PICOS framework with respect to inclusion and exclusion criteria (McKenzie et al., 2019; Richardson et al., 1995). The protocol for the study was pre-registered with PROSPERO (CRD42020215947).

### Participants

Eligible studies included child participants aged between 2 and 12 years. All child participants were screened as having social, emotional, or behavioral difficulties on psychometric assessment measures that had previously published psychometric properties. If studies were identified in which ADHD was reported, those studies were only included if psychometric evidence of child social, emotional, or behavioral difficulties was also provided. This addressed the change in diagnostic status of ADHD in the last two editions of the DSM. Studies were not excluded from the review if ADHD was present, as long as the study was primarily concerned with child behavior and not neurodevelopmental functioning. Studies were excluded from the review if the sample was comprised of less than *n* = 25 participants, was characterized by complex developmental trauma, or resided in out-of-home care.

### Interventions

Eligible studies included programs of behavioral family intervention that were delivered in an online format to parents. *Online* included all parenting programs where primary delivery was via an electronic medium. Research trials were included that specifically evaluated treatment interventions aimed to improve child and/or parent outcomes.

### Comparisons

Randomized Controlled Trials (RCTs) in which a treatment condition was compared with an inactive control condition were included in this review. Thus, control groups may only include randomization to a waitlist control group (WLC) or an alternative inactive control condition.

### Outcomes

Studies were required to psychometrically assess both child disruptive behavior and parental functioning. This assessment was required pre-treatment, post-treatment and at follow-up, and psychometric assessment measures utilised in this assessment were required to have previously published psychometric properties.

### Study Designs

Randomized controlled trials from peer-reviewed journals, published in English between 2000 and 2022 were included in the review. These dates were selected on the basis that they provided broad coverage of the delivery of programs of behavioral family intervention in an on-line format. Technical reports and dissertations were excluded from the review.

### Search Strategy

A systematic search of the electronic databases Scopus (Elsevier), PsycINFO (EBSCO), PsycArticles (EBSCO), CINAHL (EBSCO), Medline (OVID), Embase (OVID), Web of Science Core Collection, PubMed (NCIB), Cochrane Central Register of Controlled Trials and the Web of Science Core Collection was conducted. The search strategy used a multi-field format and expressed construct terms in Boolean Logic. Construct terms, including the construct key word, were applied within each field to enable consistency across databased, titles and abstracts were searched separately across databases. Table 1 presents the construct key words and construct terms used in the database search.

**Table 1:** Search terms used in the electronic database search

Construct keyword	Construct terms
Child-Behavior AND	ODD OR Conduct OR IED OR behavio* OR ADHD OR disrupt* OR opposit* OR hyperact* OR attention OR aggress* OR tantrum OR dysreg* OR emot* OR defian* OR anti-social OR disord* OR external* OR impuls* OR anger
Treatment AND	Behavio* OR family OR interven* OR parent* OR program* OR train* OR treat* OR coach* OR educat* OR psych* OR therap* OR manag* OR child* OR infan* OR you* OR juvenile OR minor OR toddler OR early year* OR preschool OR primary OR school-aged* OR dependent OR kinder* OR prep* OR mother* OR father*
Online	Internet OR net OR web OR on-line OR digital OR distance OR remote OR comput* OR etherapy OR tele-health OR eHealth OR stream* OR electronic* OR virtual

### Study Selection

Studies identified in the search were screened to remove duplicates. Studies beyond the scope of the review were identified and removed, initially by title and then by abstract. A full-text assessment of the remaining articles was undertaken independently by each author consistent with review inclusion and exclusion criteria. Interrater agreement was estimated at *k* = 0.89 using Cohen’s Kappa and disagreements were resolved through discussion.

### Data Extraction and Management

Data extracted from included studies was recorded using a data extraction form designed for this review. Extracted data included study and program details, methodological characteristics, child and parent characteristics, and psychometric assessment information. Authors of seven studies were contacted to request further details regarding parental support, module progression and the provision of additional data. The authors are immensely grateful for the assistance provided in response.

For quantitative analysis of disruptive child behavior, parent self-report data was gathered with the Eyberg Child Behavior Inventory – intensity subscale (ECBI; Eyberg, 1999), the Child Behavior Check List – externalizing subscale (CBCL; Achenbach & Rescorla, 2000) or the Conners Early Childhood Behavior Scale-defiance/aggression subscale (CECB; Conners & Goldstein, 2009). For quantitative analysis of parenting self-efficacy, parent self-report data was gathered with the Parenting Tasks Checklist – behavior self-efficacy scale (PTC; Sanders & Woolley, 2005), the Child Adjustment and Parent Efficacy Scale (CAPES; Morawska et al., 2014), the Parenting Sense of Competence scale – efficacy subscale (PSOC; Johnston & Mash, 1989) or the Toddler Care Questionnaire (TCQ; Gross & Rocissano, 1988). For quantitative analysis of parental self-regulation, parent self-report data was gathered with the Parental Anger Inventory – intensity subscale (PAI; Sedlar & Hansen, 2001) and the Parenting Scale-over-reactivity subscale (PS; Arnold et al., 1993). Finally, for quantitative analysis of parent stress, parent self-report data was gathered using the Parenting Stress Index-Short Form-parent distress subscale (PSI-SF; Abidin et al., 2006) and the Depression, Anxiety and Stress Scales – stress subscale (DASS-21; Lovibond & Lovibond, 1995).

### Risk of Bias Assessment

Methodological quality and risk of bias for included studies were assessed with the Cochrane risk of bias tool for randomized trails (Higgins & Green, 2011). Assessment of methodological quality was conducted separately by the authors independently of each other. Disagreements about methodological quality were resolved through consultation and interrater reliability was estimated at *k* = 0.91 using Cohen’s Kappa.

### Statistical Analyses

Statistical analyses were conducted using Comprehensive Meta-Analysis software version 3.0 (Borenstein et al., 2005). Hedge’s *g* effect sizes were calculated to assess within and between groups treatment on child outcomes, parenting ability, and parent outcomes. A minimum of four studies were required for an analysis to be undertaken (Bora et al., 2017; Jefferson et al., 2020) and Hedge’s *g* effect sizes were used because of the presence of studies with small sample sizes (Durlak, 2009; Ellis, 2010). Effect sizes were interpreted in accordance with Hedge’s *g* guidelines (Cohen, 1988) and random effects models were employed as the presence of random sources of error was assumed (Borenstein et al., 2010). The presence of heterogeneity was indicated by a significant *Q*-statistic (*p* < 0.05). The *I*^2^ statistic was used to estimate the percentage of heterogeneity across studies that was beyond random sample variance. A value of 0% indicated no heterogeneity; values of 0% to 40% represent limited to no heterogeneity; 30% to 60% represent moderate heterogeneity; 50% to 90% represent substantial heterogeneity and 75% to 100% represent considerable heterogeneity (Deeks et al., 2019). Funnel plots were produced to identify publication bias, and the Duval and Tweedie trim and fill method was applied to them (Duval & Tweedie, 2000). Deviations in symmetry indicated potential publication biases and, where present, the Duval and Tweedie method was used to impute the effect size of sufficient non-significant studies to address bias. Adjusted *Q* and *I*^2^ statistics derived from the Duval and Tweedie imputation were reported, as was Egger’s intercept (Egger et al., 1997) which provided an assessment of the extent to which funnel plot asymmetry was addressed through imputation. Finally, Oriwn’s failsafe N (Orwin, 1983) indicated the number of unpublished non-significant studies that would be required to lower the overall effect size below significance. Following these analyses, the potential influence of moderators on outcomes was assessed by examining the influence of continuous moderators through meta-regression and the influence of categorical moderators through sub-group analyses at the study level.

## Results

### Study Characteristics

**Fig. 1:**
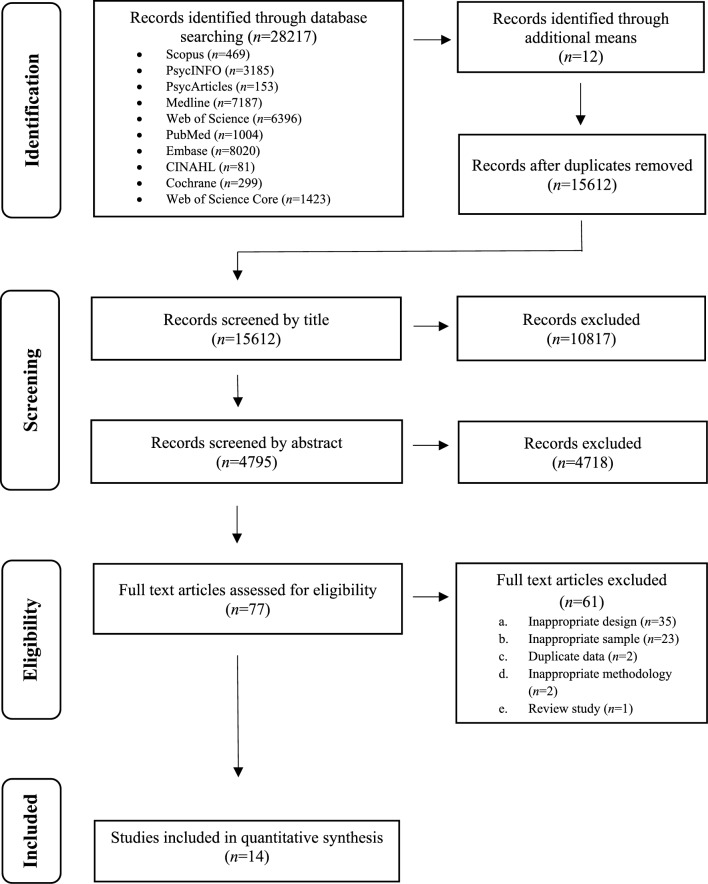
PRISMA flow chart depicting study selection process

The search strategy identified *k* = 28,217 records, with *k* = 12 additional records identified through review of reference lists of eligible studies. A PRISMA flow diagram depicting the study identification and selection process is presented in Fig. 1 and the descriptive characteristics of included studies are presented in Table 2.

**Table 2:** Descriptive characteristics of included studies

Study (Author, year, country)	Program	Duration and delivery	Design intervention/comparison	Inclusion criteria	Sample size	Child and parent demographic characteristics	Child outcomes (behavior)	Parenting ability (PSE and relational)	Parent outcomes (regulation and stress)
Baker et al. (2017) Australia	Triple P Online Brief	8 weeks Self-administered	RCT (2 groups) Intervention Internet as usual wait list control Pre/post/FU @ 9 months	Child 2–9 years old Scores in borderline clinical range or higher on SDQ	*n* = 200 Intervention (*n* = 100) Control (*n* = 100)	Child: Sex: Male TPOL = 52%, WLC = 58% *x̄* age: TPOL = 4.57 years WLC = 4.26 years Parent: Sex: TPOL = 92% female WLC = 92% female *x̄* age: TPOL = 35.74 WLC = 35.75 Relationship: TPOL = 83% married WLC = 81% married Education: TPOL = 56% university WLC = 56% university	ECBI – P/I CAPES	CAPES BCPC PCPTOS	PAI PPC DASS-21 PS
Breitenstein et al. (2016) USA	ezParent adaptation of the Chicago Parent Program	6 sequential modules + review Self-administered	RCT (2 group) Intervention Attention control condition (information already available in the public domain) Pre, post @12 and 24 weeks	Child 2–5 years old Low-income ethnic minority parents of young children Primary care pediatric clinic patients	*n* = 79 Intervention (*n* = 40) Control (*n* = 39)	Child: Sex: ezP = 57% female Control = 56.4% female Age: ezP = 36.7% 4 years Control = 35.9% 4 years Parent: Sex: ezP = 57% female Control = 56.4% female Age: ezP = 36.7% 4 years Control = 35.9% 4 years Relationship: exP = 60.8% never married Control = 53.8% never married Education: ezP = 62% College/AD Control = 59% College/AD	ECBI P/I	PQ TCQ	PSI-SF
Breitenstein et al. (2021) USA	ezParent adaptation of the Chicago Parent Program	6 sequential modules + review Self-administered	RCT (2 group) Intervention Control Health Promotion Website (comprised of information available in the public domain) Pre, and 3, 6, and 12 months post baseline	Child 2–5 years old Primary care patients	*n* = 287 Intervention (*n* = 146) Control (*n* = 143)	Child: Sex: ezP = 47.2% female Control = 51.7% female Age: ezP = *x̄* not provided Control = *x̄* not provided Parent: Sex: ezP = 90.3% mothers Control = 92.3% mothers *x̄* age: ezP = not provided Control = not provided Relationship: exP = 54.9% never married Control = 51.7% never married Education: ezP not provided Control not provided	ECBI P/I SDQ	PARYC PQ PSOC	PSI-SF
Carta et al. (2013) USA	Safe Care Planned Activities Training (PAT)	5 sessions Home visit + telephone support	3 arm RCT (inc. 2 groups) Intervention CPAT Waitlist control Pre, post, FU @ 6 months	Child 3.5–4.5 years Mother under 18 at first child’s birth across conditions High School Diploma or less across conditions	*n* = 371 Intervention (*n* = 113) WLC (*n* = 116)	Child: Sex: 56% male across conditions Age: *x̄* age 4.56 across conditions Parent: Sex: CPAT: 100% female WLC: 100% female *x̄* age: CPAT 29.02 WLC 28.66 Relationship: CPAT: 62.8% partnered WLC: 66.4% partnered Education: CPAT: 3.7% university WLC: 4.5% university	BASC-2	CBRS KIPS	PSI-SF BDI-II PS
Day and Sanders. (2018a) Australia	Triple P Online (TPOL)	8 weeks (on-line up to 4 months) Self-directed	3 arm RCT (inc. 3 groups) Intervention TPOL Waitlist control Pre, post, FU @ 5 months	Child 2–8 years To meet at least one additional socioeconomic or family risk factor associated with child social, emotional, or behavioral problems Score 5 or more on PPC	*n* = 183 TPOL (*n* = 57) Waitlist control (*n* = 60)	Child: Sex: Male TPOL = 42.10% WLC = 55.00 *x̄* age: TPOL = 3.44 WLC = 3.43 Parent: Sex: TPOL = 94.70% female WLC = 98.30% female *x̄* age: TPOL = 34.81 years WLC = 34.50 years Relationship: TPOL = 89.50% partnered WLC = 91.70% partnered Education: TPOL 65.00% university WLC 65.00% university	ECBI – P/I	RQI PDR PTC	DASS-21 PAI PS PPC
Dayand Sanders. (2018b) Australia	Triple P Online enhanced (TPOLe)	8 weeks (on-line up to 4 months Self-directed + clinical telephone support)	3 arm RCT (inc. 3 groups) Intervention TPOLe Waitlist control Pre, post, FU @ 5 months	Child 2–8 years To meet at least one additional socioeconomic or family risk factor associated with child social, emotional, or behavioral problems Score 5 or more on PPC	*n* = 183 TPOLe (*n* = 66) WLC (*n* = 60)	Child: Sex: Male TPOLe = 42.40%, WLC = 55.00 *x̄* age: TPOLe = 3.44, WLC = 3.69 Parent: Sex: TPOLe = 95.30% female WLC = 98.30% female *x̄* age: TPOLe = 35.45 years WLC = 34.50 years Relationship: TPOLe = 86.40% partnered WLC = 91.70% partnered Education: TPOLe 57.60% university WLC 65.00% university	ECBI – P/I	RQI PDR CSQ PTC	DASS-21 PAI PS PPC
Du Paul et al. (2018) USA	Project PEAK	10, weekly Self-directed + clinical telephone support	3 arm RCT (inc. 2 groups) Online intervention Waitlist control Pre, mid, post treatment	Child 3 – 5.11 years Met diagnostic criteria for one of 3 presentations of ADHD, risk for ODD	*n* = 47 Online intervention (*n* = 15) WLC (*n* = 16)	Child: Sex: Male Online = 60%, WLC = 81.25% *x̄* age: PEAK = 4.52, WLC = 4.27 Parent: Sex: PEAK = 92% female WLC = 92% female *x̄* age: PEAK not provided WLC not provided Relationship: PEAK not provided WLC not provided Education: PEAK 53.3% university WLC 62.5 university	CERS	IRP-15	PSI-SF PS
Ehrensaft et al. (2016) USA	Triple P Online (TPOL)	8, weekly Self-directed	RCT (2 groups) Online intervention Waitlist control Pre, post, FU	Child 2–6 years PSI Total Stress Scale scores exceeding the scale mean of 69 were eligible	*n* = 52 Online intervention (*n* = 26) WLC (*n* = 26)	Child: Sex: TPOL and WLC not provided Age: TPOL and WLC = 2–6 Parent: Sex: TPOL and WLC all female x̄ age: TPOL = 23.76 WLC = 24.97 Relationship: TPOL and WLC not provided Education: POL and WLC all currently attending university			PSI-SF PS
Enebrink et al. (2012) Sweden	Internet Based Parent training (PMT)	10, weekly Self-directed + non-clinical telephone support	RCT (2 groups) Intervention Waitlist control	Child 3–12 years 1 SD above the mean of the ECBI Scores above ECBI cut off (> 114)	*n* = 104 Intervention (*n* = 58) Control (*n* = 46)	Child: Sex: Male Online = 60% WLC = 81.25% *x̄* age: PEAK = 4.52, WLC = 4.27 Parent: Sex: PEAK = 92% female WLC = 92% female *x̄* age: PEAK not provided WLC not provided Relationship: PEAK not provided WLC not provided Education: PEAK 86% university WLC 93.5% university	ECBI – P/I SDQ	PPI	
Fossum et al. (2018) Canada/ Finland	Strongest Families Smart Website (SFSW)	11, weekly Self-directed + clinical telephone support	RCT (2 groups) Intervention non-treatment control Pre, 6 and 12 months post	Child 4 years Scored 5 + on the SDQ CD subscale	*n* = 464 Intervention (*n* = 232)	Child: Sex: not provided Age: 4 year children Parent: Sex: SFSW = 90% female WLC = 93% female *x̄* age: Male SFSW = 33.2 Female = 30.5 Male WLC = 31.4 Female = 29.8 Relationship: SFSW not reported WLC = not reported Education: SFSW 57.4 university WLC 58% university	CBCL 1.5–5	Barkley’s Adult AD/HD Quick Screen	DASS 21 PS
Franke et al. (2020) New Zealand	Triple P online	16, weekly Self-help	RCT (2 groups) Intervention Delayed Intervention Pre, post, FU 6 months	Child 3–4 years Met cut off criteria on WWP (≥ 14) and PACS (≥ 16)	*n* = 53 Intervention (*n* = 27) Delayed intervention (*n* = 26)	Child: Sex: Male = 71.7% across conditions Age: 3 and 4 year old children Parent: Sex: not provided *x̄* age: mothers = 35.4 across conditions fathers = 38.8 across conditions Relationship: not provided Education: 55.7% of mothers university degree	Connors EC-BEH CBS SDQ	ASRS PSDQ-A PSOC CSQ	DASS-21 PS
Porzig-Drummond et al. (2015) Australia	123 Magic	3 h 46 min of videos + tip sheets. Self-directed video-based	RCT (2 groups) Intervention Delayed Intervention Pre, post, FU 6 months	Child aged 2–10 Falls within the clinical range on the PSI-SF	*n* = 84 Intervention (*n* = 43) WLC (*n* = 41)	Child: Sex: Male 123 Magic = 62.1% WLC = 39.4% *x̄* age: 123 Magic = 5.21 WLC = 5.33 Parent: Sex: 123 Magic = 86.2% female WLC = 90.9% female *x̄* age: 123 Magic = 37.8 WLC = 38.61 Relationship: 123 Magic not reported WLC not reported Education: 123 Magic 96.6% university WLC 90.1% university	ECBI – P/I		PSI-SF DASS-21
Sanders et al. (2012) Australia	Triple P online	8, weekly Self help	RCT (2 groups) Intervention Internet as usual control (information already available in the public domain) Pre, post, FU 6 months	Child 2–9 years Elevated levels of child behavior problems on ECBI	*n* = 116 Intervention (*n* = 60) Control (*n* = 56)	Child: Sex: Male TPOL = 70% WLC = 64 x̄ age: TPOL = 4.92 WLC = 4.41 Parent: Sex: TPOL = 90% female WLC = 93% female *x̄* age: TPOL = 37.62 WLC = 37.11 Relationship: TPOL = 88 partnered WLC = 91% partnered Education: TPOLe 57% university WLC 59% university	ECBI – P/I SDQ – C/E	PTC FOS	DASS-21 PAI – P/I PS PPC – P/E
Sourander et al. (2016) Finland	Strongest Families Smart Website (SFSW)	11 weeks Self-directed parent training + weekly telephone coaching	RCT (2 groups) Intervention WLC (Website (information already available in the public domain) Pre, 6 and 12 months FU	Child aged 4 years Score of > 5 on conduct problems subscale of SDQ	*n* = 464 Intervention *n* = 232 Control *n* = 232	Child: Sex: Male SFSW = 61.20% WLC = 62.5 age: 4 years old across groups Parents: Sex: SFSW = 90% female WLC = 93% female x̄ age: Male SFSW = 33.2, Female = 30.5 Male WLC = 31.4, Female = 29.8 Relationship: SFSW not reported WLC = not reported Education: SFSW 57.4 university WLC 58% university	SDQ – C CBCL – E ICU	SOC-13	DASS-21 PS PPC

ASRS = Adult ADHD Self-Report Scale (Adler et al., 2006); BASC-II = Behavior Assessment Scale for Children-2 (Reynolds & Kamphaus, 2004); BCPC = Behavior Concerns and Parental Confidence Scale (Baker et al., 2017); CAPES = Child Adjustment and Parent Efficacy Scale (Morawska et al., 2014); CBCL = Child behavior check list (Achenbach & Rescorla, 2000); CBS = Child Behavior Scale (Ladd & Profilet, 1996); CBRS = Child Behaviour Rating Scale (Carta, 2006); CD = diagnosis of conduct disorder; CERS = Conners Early Childhood Rating Scale (Conners, 2009); Conners EC-BEH = Conners Early Childhood Behavior Scale (Conners, 2009); CSQ = Client Satisfaction Questionnaire (Sanders et al., 2012); DASS-21 = Depression, Anxiety and Stress Scale (Lovibond & Lovibond, 1995); EARL = Early Assessment Risk List-20B/21G (Augimeri et al., 2001; Levene et al., 2001); ECBI = Eyberg Child Behavior Inventory (Eyberg & Pincus, 1999); FBQ = Family Background Questionnaire (Turner et al., 2002); FOS = Adapted family observation schedule (Sanders, 2000); HSQ-M = Home Situations Questionnaire (Matthey and Barnett, 1999); ICU = Inventory of Callous-Unemotional Traits (Essau et al., 2006); IRP-15 = Intervention Rating Profile – 15 (Martens et al., 1985); KIPS = Keys to Interactive Parenting Scale (Comfort & Gordon, 2006); ODD = Oppositional Defiant Disorder; PACS = Parental Account of Child Symptoms (Taylor, Sandberg, Thorley, & Giles, 1991), PAFAS = Parenting and Family Adjustment Scale (Sanders et al., 2014); PAI = Parental Anger Inventory (Sedlar & Hansen, 2001); PARYC = Parenting Young Children (McEachern et al., 2012); PCPTOS = Parent–Child Play Task Observation System (Rusby et al., 2015); PDR = Parent Daily Report (Chamberlain & Reid, 1987); PLOC = Parent Locus of Control Scale (Campis et al., 1986); PPC = Parent Problem Checklist (Dadds & Powell, 1991); PPI = Parenting Practices Interview (Webster-Stratton, 1998; Webster-Stratton, et al., 2001); PQ = Parenting Questionnaire (Gross et al., 2004); PS = Parenting Scale (Arnold et al., 1993); PSDQ-A = Parenting Styles and Dimensions Questionnaire = (Robinson et al., 2001); PSI-SF = Parent Stress Index – Short Form (Abidin & Ablin, 1995); PSOC = Parent Sense of Competence Scale (Gibaud-Wallston & Wandersman, 1978); PSS = Parenting Satisfaction Survey (Turner et al., 2002); PTC = Parenting Tasks Checklist (Sanders & Wooley, 2005); RCT = Randomized Controlled Trial; RQI = Relationship Quality Index (Norton, 1983); SDQ = Strengths and difficulties questionnaire (Goodman, 2001); SOC-13 = Sense of Coherence Scale (Antonovsky, 1993); TCQ = Toddler Care Questionnaire (Gross & Rocissano, 1988); WLC = Waitlist Control. WWP = Werry–Weiss–Peters activity rating scale (Routh, 1978)

### Population and Sample Demographics

Fourteen studies with a total of *n* = 2,040 child participants were included in this review. Individual study sample sizes ranged from *n* = 47 to *n* = 464 children. Child participants were aged 2–12 years, with a mean age of 4.8 years across studies. The mean length of programs included in the review was 10 weeks, however program length was not reported in *k* = 3 studies. Of child study participants, 59.1% were male, and 88.1% of adult study participants were female. Studies were conducted in five different countries: Australia (*k* = 5), New Zealand (*k* = 1), Finland (*k* = 2), Sweden (*k* = 1), and the USA (*k* = 5).

### Study Design

All fourteen studies included in the review used an RCT design with an active treatment condition and an inactive control condition. Across studies, *k* = 3 (Ehrensaft et al., 2016; Enebrink et al., 2012; Fossum et al., 2018) randomized participants to one treatment group and one control group; *k* = 5 studies (Baker et al., 2017; Breitenstein et al., 2016, 2021; Sanders et al., 2012; Sourander et al., 2016) randomized participants to a control group that was characterized by the provision of information already in the public domain, and *k* = 2 studies (Franke et al., 2020; Porzig-Drummond et al., 2015) provided intervention for their control group. The remaining *k* = 3 studies (Carta et al., 2013; Day & Sanders, 2018; DuPaul et al., 2018) randomized participants to two treatment groups and one control group. Both treatment conditions offered by (Day & Sanders, 2018) met inclusion criteria for the review. Similarly, Breitenstein et al. (2021) and Breitenstein et al. (2016) both met criteria for inclusion in the review.

### Intervention Characteristics

Triple P online was used in *k* = 6 of the fourteen individual studies: k = 4 (Day & Sanders, 2018; Ehrensaft et al., 2016; Franke et al., 2020; Sanders et al., 2012) used the standard online format of Triple P, and one each used the online brief Triple P format (Baker et al., 2017), or the enhanced Triple P format (Day & Sanders, 2018). Of the remaining studies, *k* = 2 (Breitenstein et al., 2016, 2021) used EZParent, an online adaptation of the Chicago Parent Program (Breitenstein et al., 2012), *k* = 2 (Fossum et al., 2018; Sourander et al., 2016) used the Strongest Families Smart Website (SFSW) and *k* = 1 each used Safecare (Carta et al., 2013), Project PEAK (DuPaul et al., 2018), 123 Magic (Porzig-Drummond et al., 2015), or generic PMT (Enebrink et al., 2012).

### Module Progression and Completion

There was a high degree of variability in the release of session material and through that material within online programs in the review. A majority of studies, *k* = 10, released program material on a weekly or bi-weekly basis; *k* = 4 (Day & Sanders, 2018; Fossum et al., 2018; Franke et al., 2020; Sourander et al., 2016) were released on a weekly basis and *k* = 2 (Breitenstein et al., 2016, 2021) were released on a bi-weekly basis. Three studies (Baker et al., 2017; Enebrink et al., 2012; Sanders et al., 2012) released their material at less than one module every two weeks. Of the remainder (DuPaul et al., 2018) released material flexibly across 10 sessions in a sequenced manner, and (Ehrensaft et al., 2016) offered similar arrangements over an 8-week period. In all cases, program material was provided to participants in a sequenced way. Porzig-Drummond et al. (2015) provided intervention material in the form of 2 videos over 2 weeks and Carta et al. (2013) did not report on module progression.

A high degree of variability was also evident in relation to completion of programs. Two studies (Breitenstein et al., 2016; Enebrink et al., 2012) reported 100% of their participants (*n* = 40 and *n* = 58 respectively) completed treatment. Of the remainder, *k* = 6 studies had smaller sample sizes than these two; DuPaul et al. (2018) reported 88% of the *n* = 15 participants allocated to the treatment group completed treatment, Ehrensaft et al. (2016) reported 69% of the 26 participants allocated to the treatment group completed treatment, Porzig-Drummond et al. (2015) reported 79% of the *n* = 43 participants allocated to treatment completed treatment and Franke et al. (2020) reported 88% of the *n* = 53 participants allocated to treatment completed treatment. Day and Sanders (2018) reported 70% of the *n* = 57 participants in the TPOL and 76% of the *n* = 66 participants in the TPOLe condition completed treatment. (Sanders et al., 2012).

The larger studies included in the review all recruited over one hundred participants. Baker et al. (2017) reported recruiting *n* = 100 participants of whom 98% completed treatment, and Carta et al. (2013) reported recruiting *n* = 113 participants however did not report on treatment completion. (Breitenstein et al., 2021) recruited *n* = 146 into treatment of whom 58% completed treatment and both (Fossum et al., 2018) and (Sourander et al., 2016) reported recruiting *n* = 232 however only (Sourander et al., 2016) reported the successful completion of treatment, in that case of 59% of participants.

### Psychometric Assessment

Included studies utilized a range of methods to determine if participants met inclusion criteria. The most common method was psychometric assessment. Two measures were predominant, the ECBI (Eyberg, 1999) was used by *k* = 7 studies (Baker et al., 2017; Breitenstein et al., 2016, 2021; Day & Sanders, 2018; Enebrink et al., 2012; Porzig-Drummond et al., 2015; Sanders et al., 2012). The SDQ was used by *k* = 5 studies (Breitenstein et al., 2021; Enebrink et al., 2012; Franke et al., 2020; Sanders et al., 2012; Sourander et al., 2016). In each case, studies reported using subscales or associated metrics to establish inclusion. One study each identified and used cutoffs on additional psychometric assessment measures including the PPC (Day & Sanders, 2018), the Connors (DuPaul et al., 2018), the PSI (Ehrensaft et al., 2016), the WWP and the PACS (Franke et al., 2020). The remainder of studies (*k* = 5) reported relying on socioeconomic or family risk factors for inclusion in their study prior to undertaking psychometric assessment to establish the presence of behavioral difficulties (e.g., Breitenstein et al., 2016; Carta et al., 2013). In addition to psychometric assessment, (*k* = 2) studies reported assessing child disruptive behavioral characteristics against DSM-4 or DSM-5 criteria (Day & Sanders, 2018; DuPaul et al., 2018), however, diagnosis was not sought in either study.

### Parental Support

Additional forms of support were provided to parents participating in some of the programs delivered in the included studies. These included the provision of a face to face initial session in *k* = 5 studies (Breitenstein et al., 2016; Breitenstein et al., 2021; Carta et al., 2013; DuPaul et al., 2018; Ehrensaft et al., 2016); the provision of therapeutic telephone support in *k* = 7 studies (Carta et al., 2013; Day & Sanders, 2018; Ehrensaft et al., 2016; Enebrink et al., 2012; Fossum et al., 2018; Franke et al., 2020; Sourander et al., 2016); the provision of therapeutic written support in *k* = 7 studies (Breitenstein et al., 2016, 2021; Ehrensaft et al., 2016; Enebrink et al., 2012; Fossum et al., 2018; Porzig-Drummond et al., 2015; Sanders et al., 2012); and text prompts in *k* = 7 studies (Breitenstein et al., 2016, 2021; Carta et al., 2013; Day & Sanders, 2018; Ehrensaft et al., 2016; Porzig-Drummond et al., 2015; Sanders et al., 2012).

### Quality Assessment

The methodological quality of the studies retained for review was assessed against the Cochrane Collaboration’s tool for assessing risk of bias in RCTs (Higgins & Green, 2011). High risk for performance bias and detection bias was identified in most studies. This was due largely to limitations in blinding of study personnel and participants, and potential bias was identified from the use of self-report outcome measures. This, together with the identification of other potential forms of bias, resulted in an overall rating of high risk for all studies. A summary of the methodological quality of studies retained for review is presented in Table 3, and cumulative ratings of study quality as assessed using the Cochrane risk of bias tool for randomized trails (Higgins & Green, 2011) is presented in Table 4.

**Table 3:** Summary of methodological quality and risk of bias for included studies as assessed using the Cochrane risk of bias tool for randomized trails (Higgins & Green, 2011)

Study (author, date)	Random sequence allocation (selection bias)	Allocation concealed (selection bias)	Blinding of study personnel (performance bias)	Blinding of outcome assessment (detection bias)	Incomplete outcome data addressed (attrition bias)	Free of selective reporting (selective reporting)	Free of other bias	Overall rating
Baker et al. (2017)	Low risk	Low risk	High risk^c^	High risk^d^	Low risk	Low risk	High risk^ik^	High risk
Breitenstein et al. (2016)	Low risk	Low risk	High risk^c^	High risk^d^	Low risk	Low risk	High risk^d^	High risk
Breitenstein et al. (2021)	Low risk	Low risk	High risk^c^	High risk^d^	Low risk	Low risk	High risk^d^	High risk
Carta et al. (2013)	Unclear^a^	Unclear^b^	High risk^b^	Low risk	Low risk	Low risk	High risk^l^	High risk
Day and Sanders (2018)	Low risk	Low risk	High risk^c^	High risk^d^	High risk^f^	Unclear^h^	High risk^ik^	High risk
Day and Sanders (2018) (TPOLe)	Low risk	Low risk	High risk^c^	High risk^d^	High risk^f^	Unclear^h^	High risk^ik^	High risk
Du Paul et al. (2018)	Unclear^a^	Unclear^b^	High risk^c^	High risk^d^	Unclear^g^	Unclear^h^	High risk^km^	High risk
Ehrensaft et al. (2016)	High risk^a^	High risk^b^	High risk^c^	High risk^d^	Low risk	Low risk	High risk^l^	High risk
Enebrink et al. (2012)	Low risk	Low risk	High risk^c^	High risk^c^	High risk^g^	Unclear^h^	High risk^kl^	High risk
Fossum et al. (2018)	Low risk	Unclear^b^	High risk^c^	High risk^c^	Low risk	Low risk	High risk^di^	High risk
Franke et al. (2020)	Low risk	Unclear^b^	High risk^c^	High risk^d^	Low risk	Low risk	High risk^ikm^	High risk
Porzig-Drummond et al. (2015)	Low risk	Low risk	Unclear^c^	Unclear^c^	Low risk	Low risk	High risk^dh^	High risk
Sanders et al. (2012)	Low risk	Unclear^b^	High risk^c^	High risk^d^	Low risk	Unclear^h^	High risk^ijk^	High risk
Sourander et al. (2016)	Low risk	Low risk	High risk^c^	High risk^c^	Low risk	Low risk	High risk^i^	High risk

Assessment was conducted according to the Cochrane Collaboration’s tool for assessing risk of bias in RCTs (Higgins & Green, 2011)

^a^Did not provide adequate detail on method of randomization to establish low risk of selection bias during allocation stage

^b^Did not provide adequate detail on allocation concealment

^c^Ability to adequately blind participants, study personnel, and outcome assessment in psychological treatments is restricted

^d^Outcome measures were mostly parental self-report with ingrained detection bias

^e^Analyses for child and parent outcomes conducted as intent-to-treat analyses (ITT)

^f^ > 20% participant loss and no ITT

^g^Did not provide adequate detail to establish low risk of attrition bias

^h^Difficulty in accurately assessing selective reporting due to nil study registration, retrospective registration or nil protocol publication

^i^Conflict of interest identified

^j^Funding source or whether there is a conflict of interest was not disclosed

^k^Sample higher than average SES

^l^Homogenous sample population will low generalisability

^m^Small sample size

**Table 4:**
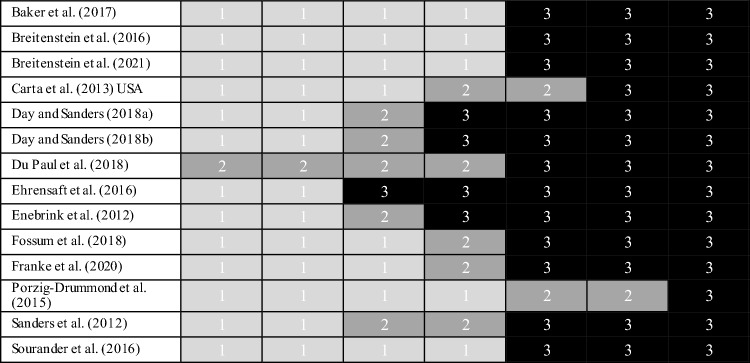
Cumulative ratings of study quality and risk of bias as assessed using the Cochrane risk of bias tool for randomized trails (Higgins & Green, 2011): 1 = low risk; 2 = unclear risk; 3 = high risk

^a^Siteâ€‰=â€‰Site 1 includes UAI 1â€‰=â€‰"Dr. Isaac Cohen AlcahÃ©" UAI and Rodolfo Robles Hospital. Site 2 includes UAI 2â€‰=â€‰"Dr. Carlos Rodolfo MejÃ­a" UAI and Roosevelt Hospital

^b^McNemar exact test was used when comparing LAM test alone and LAM test after Î±-mannosidase treatment

### Effect on Child Outcomes

Of the fourteen studies that were included in the review, *k* = 10 provided data that facilitated analysis of pre- follow-up within-group effects on child externalising behavior. A large and significant overall treatment effect was evident across studies (Hedge’s *g* = 0.83, *SE* = 0.17, *95%CI* = 0.50–1.16*, p* = 0.00). Within-group treatment effects for child externalising behavior represented decreases in parent report of child externalising behavior and ranged from small to large (Hedge’s *g* = 0.19–2.63). Large and significant heterogeneity was evident (*Q*(9) = 85.07, *p* = 0.00) and a large proportion of identified heterogeneity across studies was beyond random sample variance (*I*^2^ = 89.42). Duval and Tweedie’s (2000) trim and fill method indicated asymmetry and so *k* = 3 studies were imputed to address bias. As a result, the point estimate increased (*Q*(9) = 226.80, *95%CI* = 0.70–1.49). Egger’s intercept (Egger et al., 1997) was non-significant indicating no remaining publication bias (*α* = 3.39, *95%CI* =  − 3.14 to 9.93*, t* = 1.20,* p* = 0.27). Orwin’s failsafe N (Orwin, 1983) indicated *k* = 500 additional studies would be required to bring the overall effect below significance.

Post-intervention between groups treatment effects on child externalising behavior were assessed in *k* = 10 of the included studies. Moderate and significant between groups treatment effects were evident (Hedge’s *g* = 0.42, *SE* = 0.12, *95%CI* = 0.20–0.65*, p* = 0.00*)* and individual effect sizes ranged from zero to large (Hedge’s *g* = 0.00–0.96). Effect sizes represent reductions in parent report of child externalising behavior compared to control, and results indicated moderate, significant heterogeneity (*Q*(9) = 37.68, *p* = 0.00). A large proportion of heterogeneity across studies was beyond random sample variance (*I*^2^ = 76.11). Duval and Tweedie’s (2000) trim-and-fill method indicated *k* = 1 imputed study was required to address publication bias. As a result of imputation, the point estimate decreased marginally (Hedge’s *g* = 0.40*, 95%CI* = 0.20–0.61) and heterogeneity increased to a similar extent (*Q*(9) = 46.78, *95%CI* = 0.13–0.59). Egger’s intercept (Egger et al., 1997) was non-significant indicating no publication bias (*α* = 2.54, *95%CI* =  − 1.28 to 6.37*, t* = 1.53*, p* = 0.167*).* Orwin’s failsafe N (Orwin, 1983) indicated that *k* = 118 studies with zero effects would be required to reduce the overall effect below significance.

### Effect on Parenting Ability

Of the fourteen studies included in the review, *k* = 8 provided data that allowed analysis of pre- follow-up within-group treatment effects on parenting self-efficacy with effects representing increases in parent report of parenting confidence and ability. Random effects models revealed a large and significant treatment effect on parenting self-efficacy across studies (Hedge’s *g* = 0.86, *SE* = 0.20, *95%CI* = 0.47–1.25*, p* = 0.00). Individual study within-group effects for parenting self-efficacy ranged from small to large (Hedge’s *g* = 0.16–1.8). Moderate and significant heterogeneity was evident (*Q*(7) = 48.28, *p* = 0.00) and a large proportion of identified heterogeneity across studies was beyond random sample variance (*I*^2^ = 87.57). Duval and Tweedie’s (2000) trim and fill method indicated no additional studies were required to be imputed into the analysis. Egger’s intercept (Egger et al., 1997) did not differ significantly from zero indicating limited asymmetry in the funnel plot (*α* = 6.40, 95%*CI* =  − 2.50 to 15.29*, t* = 1.85*, p* = 0.12*)* and the original overall effect size and level of publication bias were maintained. Orwin’s failsafe N (Orwin, 1983) indicated *k* = 229 additional studies with zero effect would be required to bring the overall effect under significance.

Data appropriate to the analysis of post-intervention between-group effects on parenting self-efficacy was derived from *k* = 7 studies included in the review. Random effects models revealed a moderate and significant overall treatment effect across studies (Hedge’s *g* = 0.37, *SE* = 0.14, *95%CI* = 0.10–0.63*, p* = 0.01*)*. Individual between-group treatment effects for parenting self-efficacy represented increases in parent report of parenting skills and ability compared to control and ranged from small to large (Hedge’s *g* = 0.02–0.86). Moderate and significant heterogeneity was evident (*Q*(6) = 23.34* p* = 0.00) and a large proportion of identified heterogeneity across studies was beyond random sample variance (*I*^2^ = 74.29). Duval and Tweedie’s (2000) trim and fill method indicated *k* = 2 studies should be imputed to address publication bias. Egger’s intercept (Egger et al., 1997) was non-significant indicating a lack of asymmetry in the funnel plot (*α* = 5.23, *95%CI* = 0.83–11.30*, t* = 2.22*, p* = 0.08*).* A reduction in effect size (Hedge’s *g* = 0.21, *95%CI* =  − 0.06 to 0.49), and an increase in heterogeneity (*Q*(6) = 39.13, *p* = 0.00) resulted from imputation. Orwin’s failsafe N (Orwin, 1983) indicated *k* = 39 additional studies with zero effect would be required to bring the overall effect below alpha.

Of the fourteen studies that were included in the review, *k* = 4 provided data that allowed analysis of pre- follow-up within-group effects on parent–child relational development within the context of family systems environments. A small and non-significant within groups pooled effect was evident (Hedge’s *g* = 0.68, *SE* = 0.08, *95%CI* =  − 0.09 to 0.30,* p* = 0.41*).* Individual study effects ranged from Hedge’s* g* = 0.02–0.20 and small and non-significant heterogeneity was evident (*Q*(3) = 0.86, *p* = 0.86). The *I*^2^ statistic indicated no heterogeneity across studies beyond that attributable to random sample variance (*I*^2^ = 0.00). Duval and Tweedie’s (2000) trim and fill method indicated *k* = 1 study should be imputed to address publication bias identified in favor of a bias toward positive findings. Egger’s intercept (Egger et al., 1997) was not statistically significant, (*α* = 1.28*, 95%CI* =  − 3.29 to 5.85*, t* = 1.20*, p* = 0.35). The addition of *k* = 1 study resulted in a reduction in effect size (Hedge’s *g* = 0.05, *95%CI* =  − 0.10 to 0.20), and an increase in heterogeneity (*Q*(3) = 1.25, *p* = 0.00).

The same *k* = 4 studies were included in between groups analyses of treatment effects on parent–child relational development. A small and non-significant between groups pooled effect was evident (Hedge’s *g* = 0.07, *SE* = 0.08, *95%CI* =  − 0.10 to 0.23,* p* = 0.42) and a range of individual study effects were identified (Hedge’s *g* = 0.02–0.21). Small and non-significant heterogeneity was evident between groups (*Q*(3) = 0.74, *p* = 0.86). The *I*^2^ statistic indicated limited dispersion beyond random sample variance (*I*^2^ = 0.00). Duval and Tweedie’s (2000) trim and fill method indicated that *k* = 1 study should be imputed to address asymmetry in the funnel plot. Following that, Egger’s intercept (Egger et al., 1997) was statistically non- significantly (*α* = 0.77*, 95%CI* =  − 4.95to 6.48*, t* = 0.58*, p* = 0.62) indicating publication bias was addressed through imputation. The addition of *k* = 1 study resulted in a marginal increase in effect size (Hedge’s *g* = 0.10, 95%*CI* =  − 0.03 to 0.23), and an increase in heterogeneity (*Q*(3) = 1.28, *p* = 0.00).

### Effect on Parent Outcomes

#### Parent regulation—Anger

Of the fourteen studies that were included in the review, *k* = 7 provided data that allowed analysis of pre- follow-up within-group effects on parent regulation of anger. Random effects models revealed a moderate yet significant overall treatment effect within studies (Hedge’s *g* = 0.40, *SE* = 0.06, *95%CI* = 0.24–0.48*, p* = 0.00*)*. Individual treatment effects for parenting knowledge and skills had a broad range (Hedge’s *g* = 0.26–0.66) with effects representing decreases in parent self-report of anger. Small and statistically non-significant heterogeneity was present (*Q*(5) = 3.40, *p* = 0.64), and the *I*^2^ statistic indicated no dispersion beyond random sample variance (*I*^2^ = 0.00). Duval and Tweedie’s (2000) trim and fill method resulted in *k* = 3 studies being imputed to address publication bias. As a result, the point estimate decreased and the *Q* value increased (Hedge’s *g* = 0.30, *Q* = 7.83). Egger’s intercept (Egger et al., 1997) was statistically significant indicating the presence of publication bias (*α* = 2,18*, 95%CI* = 1.50–2.85*, t* = 8.95*, p* = 0.00*).* Orwin’s failsafe N (Orwin, 1983) indicated *k* = 53 additional studies with zero effect would be required to bring the overall effect below significance.

Post-intervention between groups treatment effects on parent regulation of anger were assessed in *k* = 6 of the included studies. Random effects models revealed a moderate and statistically significant between groups treatment effect across studies (Hedge’s *g* = 0.50, *SE* = 0.15, *95%CI* = 0.20–0.79*, p* = 0.00*).* Individual effect sizes ranged from small to large (Hedge’s *g* = 0.21–1.80) and represent post treatment reductions in parent anger. Moderate and significant heterogeneity was evident (*Q*(7) = 24.23, *p* = *0.00*), and the *I*^2^ statistic indicated dispersion beyond random sample variance (*I*^2^ = 79.37). Inspection of the funnel plot indicated the presence of publication bias in favor of studies reporting reductions in parent anger. Duval and Tweedie’s (2000) trim and fill method resulted in the imputation of *k* = 2 studies, and, as a result, the overall effect size increased (Hedge’s *g* = *0.70, 95%CI* = *0.35–1.05)* as did heterogeneity (*Q* = 86.58). Egger’s intercept (Egger et al., 1997) was significantly different from zero indicating the presence of asymmetry in the funnel plot (*α* = 4.15*, 95%CI* =  − 1.09 to 9.09*, t* = 2.20,* p* < 0.09*).* Orwin’s failsafe N (Orwin, 1983) indicated* k* = 65 additional studies with zero effect would be required to bring the overall effect below significance.

#### Parent Regulation—Stress

Of the fourteen studies that were included in the review, *k* = 9 provided data that facilitated analysis of pre- follow-up within-group effects on parent regulation of stress. Random effects models indicated a moderate and significant overall treatment effect (Hedge’s *g* = 0.41, *SE* = 0.09, *95%CI* = 0.23–0.60*, p* = 0.00). Individual treatment effects for parenting knowledge and skills had a broad range (Hedge’s *g* = 0.07–1.11) with effects representing decreases in parent self-reported levels of stress. Heterogeneity was small-moderate and significant (*Q*(8) = 20.63, *p* = 0.01). Dispersion was large and beyond that anticipated to result from random sample variance (*I*^2^ = 61.23). Duval and Tweedie’s (2000) trim and fill method did not indicate the imputation of any studies. As a result, the adjusted effect size remained constant. Egger’s intercept (Egger et al., 1997) was just statistically non-significant indicating the absence of asymmetry in the funnel plot (*α* = 2.66, 95% CI =  − 0.10 to 5.41, *t* = 2.28, *p* = 0.05*).* Orwin’s failsafe N (Orwin, 1983) indicated* k* = 104 additional studies with zero effect would be required to bring the overall effect below alpha.

Post-intervention between groups treatment effects on parent regulation of stress were assessed in *k* = 10 of the included studies. Random effects models revealed a moderate and statistically significant between groups treatment effect across studies (Hedge’s *g* = 0.28, *SE* = 0.05, *95*% CI = 0.19–0.38, *p* = 0.00*).* Individual effect sizes ranged from small to large (Hedge’s *g* = 0.04–0.58). Effect sizes represent post treatment reductions in parent anger and inspection of the funnel plot suggested the presence of publication bias in favor of studies reporting reductions in stress. Heterogeneity was small and non-significant (*Q*(9) = 5.93, *p* = 0.75). The *I*^2^ statistic indicated no dispersion beyond random sample variance (*I*^2^ = 0.00). Duval and Tweedie’s (2000) trim and fill method indicated that *k* = 4 studies should be imputed; this resulted in a reduction in the effect size (Hedge’s *g* = 0.21) and an increase in heterogeneity (*Q*(9) = 13.97). Egger’s intercept (Egger et al., 1997) was nonsignificant (*α* = 0.50, 95% CI =  − 1.24 to 2.24, *t* = 0.66, *p* = 0.53*)* indicating a lack of publication bias. Orwin’s failsafe N (Orwin, 1983) indicated* k* = 70 additional studies with zero effect would be required to bring the overall effect below alpha.

A summary of the within and between groups treatment effects, together with heterogeneity data, is presented in Table 5. The strongest effects were demonstrated within and between groups for child behaviour and parenting self-efficacy. Moderate and statistically significant effects were also demonstrated within and between groups for the parent emotion regulation analyses. Statistically non-significant treatment effects were evident for parent–child relational development. Significant heterogeneity was indicated in child behaviour and parenting self-efficacy analyses, and in the between groups analysis for parent anger and the within groups analysis for parent stress. By contrast, significant heterogeneity was not evident in either parent–child relational development analysis, the within groups parent emotion regulation-anger analysis or the between groups parent emotion regulation–stress analysis.

**Table 5:** Within and between groups effects of intervention on outcomes

	*n*-studies	Hedges-*g*	*SE*	95% CI	*z* value	Q	*I*^2^
Child behavior within	10	0.83***	0.17	0.50–1.16	4.99	*Q*(9) = 85.07***	89.42
Child behavior between	10	0.42***	0.12	0.20–0.65	3.67	Q(9) = 37.68***	76.11
Parenting self-efficacy within	8	0.86***	0.20	0.47–1.25	4.28	Q(7) = 48.28***	87.57
Parenting self-efficacy between	7	0.37***	0.14	9.10–0.63	2.71	Q(6) = 23.34***	74.29
Parent–child relational development within	4	0.68	0.08	− 0.09 to 0.30	0.83	Q(3) = 0.86 ns	0.00
Parent–child relational development between	4	0.07	0.08	− 0.10 to 0.23	0.81	Q(3) = 0.74 ns	0.00
Parent emotion regulation—anger within	7	0.40***	0.06	0.24–0.48	5.85	Q(5) = 3.40 ns	0.00
Parent emotion regulation—anger between	6	0.50***	0.15	0.20–0.79	6.49	Q(7) = 24.23***	79.37
Parent emotion regulation—stress within	9	0.41***	0.09	0.23–0.60	4.37	Q(8) = 20.63***	61.23
Parent emotion regulation—stress between	10	0.28***	0.05	0.19–0.38	5.68	Q(9) = 5.93 ns	0.00

### Moderation Analyses

The potentially moderating influence of variables associated with treatment outcome were assessed in two ways. Variables that were represented by continuous data were assessed with meta-regression; categorical variables were assessed with sub-group analyses undertaken at the study level. The potential moderators that were identified in this review and explored in moderation analyses are presented in Table 6.

**Table 6:** Potential continuous and categorical moderators of treatment outcome identified across the included studies

Study	Potential continuous moderators							Potential categorical moderators			
	Number of Sessions	Number experimental group	Age experimental group	% Male experimental group	FU months	Parent Sex (f)	Parent Edu (Uni)	F2F Initial Session	Therapeutic telephone support	Therapeutic written support	Text prompts
Baker et al. (2017)	8	200	4.54	52	36	92	56	0	0	0	0
Breitenstein et al. (2016)	6	40	4	43	24	57	62	1	0	1	1
Breitenstein et al. (2021)	6	287		53	52	90		1	0	1	1
Carta et al. (2013)	5	113	4.56	56	26	100	3.7	1	1	0	1
Day and Sanders (2018)	8	57	3.44	42.1	22	94.7	65	0	1	0	1
Day and Sanders (2018) (TPOLe)	8	66	3.44	42.4	22	95.3	57	0	1	0	1
DuPaul et al. (2018)	10	15	4.52	60		92	29	1	1	0	0
Ehrensaft et al. (2016)	8	26	4					1	1	1	1
Enebrink et al. (2012)	7	58	4.52	60	26	92	86	0	1	1	0
Fossum et al. (2018)	12	232	4		52	90	57.4	0	1	1	0
Franke et al. (2020)	8	27	3.5	72	26	100	55.7	0	1	0	0
Porzig-Drummond et al. (2015)	3 h 46 min	43	5.21	62	26	86.2	96.6	0	0	1	1
Sanders et al. (2012)	8	60	4.92	70	26	90	57	0	0	1	1
Sourander et al. (2016)	11	232	4	61	52	90	57.4	0	1	0	0

#### Continuous Moderators

Of the potential continuous moderators identified, three were found to significantly moderate study outcomes. The number of sessions provided to parents significantly moderated parent self-efficacy within groups (*β* = 0.44, *SE* = 0.09, *p* = 0.00) such that the greater the number of sessions that better parents report of their effectiveness. The number of parent participants in each study identified as a moderator in terms of child outcomes within groups (*β* =  − 0.00, *SE* = 0.00, *p* = 0.03), parents report of their effectiveness between groups (*β* =  − 0.00, *SE* = 0.09, *p* = 0.00) and parenting stress within groups (*β* =  − 0.00, *SE* = 0.00, *p* = 0.00), such that the greater the number of participants the smaller the effect. Level of parent education was also found to moderate child outcomes within groups such that the greater the level of education the greater the effect size (*β* = 0.02, *SE* = 0.01, *p* = 0.02).

#### Categorical Moderators

Categorical moderators were also assessed for their influence on outcomes. The inclusion of a face-to-face initial session with participants was found to positively and significantly influence within and between groups child outcomes (*Q*(1) = 8.29, *p* = 0.00; *Q*(1) = 8.28, *p* = 0.00), within and between groups parent self-efficacy outcomes (*Q*(1) = 6.44, *p* = 0.01), (*Q*(1) = 34.47, *p* = 0.00 respectively), and parents within groups parent stress (*Q*(1) = 4.46, *p* = 0.04), such that greater effect was evident with the inclusion of an initial face to face session. The provision of therapeutic clinical support in addition to material provided in session was also found to significantly moderate parent stress between groups (*Q*(1) = 4.46, *p* = 0.04), such that the provision of support increased effect between groups.

## Discussion

This systematic review and meta-analysis sought to synthesize the findings of prospective longitudinal research into the effectiveness of online programs of behavioral family intervention. The review identified *k* = 14 prospective longitudinal studies published between 2000 and 2022 that reported on the effectiveness of online programs. Child participants in the studies were aged between 2 and 12 years and program data was required to have been reported pre- and post-treatment, and at follow-up. Three distinct domains of function were assessed in the review. First, child treatment outcomes were assessed as the potential for programs to demonstrate change in disruptive child behavior. Second, parenting ability was assessed as the potential for programs to facilitate change in parenting self-efficacy, and to result in change in the quality of parent–child relational development. Finally, parent outcomes were assessed as change in parents’ capacity to regulate emotion in the form of stress and to control behavior in the form of anger. Review findings indicated that outcomes may be moderated by some continuous and categorical variables.

### Child Behavior

Online programs of behavioral family intervention included in the review demonstrated the greatest benefit on disruptive child behavioral outcomes. A broad range of treatment effects were evident both within and between groups. The review identified a large and statistically significant pre- follow-up within groups treatment effects across studies and a moderate and statistically significant between groups post treatment effect across studies. Large and statistically significant heterogeneity was present within groups and moderate and statistically significant heterogeneity was present between groups. The moderating effects of two variables were found to influence child behavioral outcomes within groups: smaller effects were identified in studies with greater numbers of participants, and the inclusion of an initial face-to-face session improved within groups child outcomes. Between groups, level of parent education, the inclusion of an initial face to face session and the inclusion of pre-session text prompts statistically significantly improved outcomes.

### Parenting Ability

Online programs of behavioral family intervention also had a beneficial effect on parenting ability, albeit to a lesser extent than that demonstrated for child behavior. Large and statistically significant within groups treatment effects and moderate and statistically significant between groups treatment effects were evident on parent report of self-efficacy. In both cases, moderate and significant heterogeneity was present. The moderating effects of four variables were evident on outcome. Within groups, outcomes were improved by more sessions and the inclusion of an initial face-to-face session. Between groups, smaller effects were identified in studies with greater numbers of participants, however they were also improved with the inclusion of an initial face-to-face session.

Online programs of behavioral family intervention included in this review were less able to demonstrate beneficial treatment effects on parent–child relational quality. Only *k* = 4 studies provided data that assessed parent–child relational quality, suggesting it may not be widely regarded as key to program efficacy. Individual studies included in the review showed a range of treatment effects, however small and non-significant overall within and between groups treatment effects were evident. Small and non-significant heterogeneity was also demonstrated both within and between groups. Moderation analyses did not reveal any statistically significant continuous or categorical moderators on outcome.

### Parent Outcomes

Online programs of behavioral family intervention had beneficial treatment effects on parent regulation of anger. The review identified moderate and statistically significant within and between groups treatment effects*.* Moderate and statistically significant heterogeneity was present both within and between groups. No moderating effects of treatment outcome were identified for either within or between groups outcomes.

Online programs of behavioral family intervention also had a beneficial effect on parent regulation of stress. The review identified a moderate and statistically significant within groups treatment effect and a small and statistically significant between groups treatment effect*.* Small yet statistically significant heterogeneity was present within groups however in the between groups it was small and non-significant. Within groups, smaller treatment effects were identified in studies with greater numbers of participants, and the inclusion of an initial face-to-face session improved treatment outcomes. Between groups, the addition of therapeutic telephone support was also found to improve treatment outcomes.

## Conclusion

Traditionally, the primary purpose of online programs of behavioral family intervention has been to demonstrate change in child behaviour. As research has increased knowledge about the mechanisms that return such change for children, the scope of programs of behavioral family intervention has broadened. In addition to understanding the importance of operant principles and the potential of social learning (Bandura, 1977; Skinner, 1969), we now have increased knowledge about the fundamental importance of parent–child relational capacity (Burke et al., 2002; Kjøbli et al., 2023). Child development is dependent on experience, and developmental experience is gained within relationships (Pollak, 2003). Parent–child relationships underpin behavioral family systems (Geeraerts et al., 2021) and are essential to child social, emotional, and behavioral functioning (Kjøbli et al., 2023). Few programs included in this review reported on change in parent–child relational quality, and none were identified as including treatment components specifically directed at influencing those relationships. Potential therefore exists to increase the effectiveness of online programs of behavioral family intervention by developing parent–child relational quality.

A second way that knowledge about parent–child relational development has potential to inform programs of behavioral family intervention is through parents’ own capacity to regulate emotion. Parents’ emotion regulation influences child development (Crespo et al., 2017). Toddlers whose relational experience is characterised by regulated emotion have better developmental, regulatory, and relational outcomes than toddlers whose developmental experience is characterised by dysregulation or reactive emotion (Crespo et al., 2017; Zimmer-Gembeck et al., 2017). Children experience their caregivers’ regulation, and their own regulatory systems are crafted in kind (Feldman, 2016; Morris et al., 2017). For instance, evidence suggests that engagement by a caregiver whose sympathetic nervous system is aroused will engender sympathetic arousal in a child in response. Repeated interactions characterised by sympathetic arousal in a caregiver will result in the relationally based organization of neural networks in the child, and their subsequent manifestation in cognitive and behavioural terms (Feldman, 2016). In practice, emotion regulation represents the continual interplay between sympathetic and parasympathetic processes, temperamental vulnerabilities and strengths, and relationally driven experience that contributes to bio-behavioural, relational, and cognitive development (Feldman, 2016). These processes revolve primarily around the capacity for the expression and regulation of emotion within the family system, the development of child regulation as a result of processes of parent–child co-regulation (Schore, 2005; Somers et al., 2021), and the extent and manner in which regulation/dysregulation influences/is influenced by relational transactions within that system (Hajal & Paley, 2020; Morris et al., 2017). It is for these reasons that current programs of preventative intervention for child social, emotional and behavioural difficulties that are developed on the basis of current evidence consider parental emotion regulation as a key target of intervention in addressing family systems that inadvertently engage undesirable child behaviour (McAloon & Lazarou, 2019).

Successfully translating the regulation of parent emotion is, perhaps, even more important in online programs of behavioral family intervention when assisting parents to respond to child social, emotional, and behavioral functioning they want to see decrease. Often articulated as *bad* behavior, parental dysregulation may accompany parent engagement of children in response to behavior parents want to see less of. Successfully imparting regulatory strategies to reduce the contingently reinforcing potential of parental engagement of undesirable child behavior may assist in reducing that behavior once treatment components directed at increasing desirable child behavior have been delivered. Thus, the developmental implications of effective interventions directed at achieving parent emotion regulation in the context of child observational learning, and in addressing undesirable child behavior, are potentially significant.

Finally, the moderators of treatment outcome reported in this review have important implications for online programs. The quality of treatment-based research, particularly with respect to sample size, is already extensively documented. Underpowered studies have potential to misreport effects and therefore potentially bias evidence. The estimation of a number of sessions that can successfully embed change, together with the addition of an initial face-to-face session, and the provision of text reminders, have demonstrated potential to improve treatment outcomes. Certainly, families will continue to present with a range of educational backgrounds and qualifications. These characteristics assist in aligning our understanding of manualized programs with the knowledge that different clients will benefit from differing translations of program content and research evidence. Ultimately, this review assists in reminding us that manualized programs of behavioral family intervention that must fit the client. It is seldom the client who should fit the manual.

## Limitations and Future Directions

This review has some noteworthy limitations and some key implications for future work in the area. To review as homogeneous a range of research as possible, the review constrained the included studies to prospective longitudinal research that included randomization and inactive comparison groups. This aim was largely achieved; however, it may be that important research was not reviewed because of stringent inclusion/exclusion criteria. For instance, it is important to acknowledge that family behavioral systems, and therefore family behavior, functions consistent with the cultural origins of those systems and the cultural influences on those systems. The review may be limited in its ability to identify research culturally diverse research important to our understanding about generalizing findings to culturally diverse populations and societies.

The review also sought to assess the effectiveness of online programs of behavioral intervention. It is timely that a review of this body of work is undertaken. Online programs of behavioral intervention are currently extensively used and the importance of parent relational and regulatory behavior for optimal child development cannot be understated. The authors’ intention in limiting the review to online programs is not to suggest that there are substantial theoretical or empirical differences between online and face-to-face modes of delivery. Rather, the intention was to acknowledge that the online delivery of programs of behavioral family intervention may currently be regarded as a mode of treatment delivery in its own right.

The review holds significant implications for the continued development of online programs of behavioral family intervention. These revolve around the importance of nurturing parent–child relational characteristics that facilitate optimal child development (Kaehler et al., 2016). Family environments may be regarded as functional behavioral systems that are characterised by the qualities of the interpersonal relationships of which they are comprised (Kjøbli et al., 2023). It has also been acknowledged that relational interactions within family environments are transactional, and that transactional processes in the context of behavioral family systems influence child development and adaptation (Kochanska et al., 2019; Newton et al., 2014). In a reciprocal fashion, they also have the potential to influence parent functioning and parenting behavior. It is of central importance to acknowledge that one of the functions of parents within behavioral family systems is to nurture the transactional relationships within those systems. Explicitly articulating and assessing this within online programs of behavioral family intervention has potential to convey to parents the potential they hold in facilitating child social emotional and behavioral change (Kjøbli et al., 2023; Leijten et al., 2019).

## Summary

Results from this study inform the future of online programs of behavioral family intervention. We reviewed current prospective longitudinal research about online programs of behavioural family intervention in terms of child outcomes, parenting ability and parent outcomes. The review synthesized findings of *k* = 14 studies published between 2000 and 2022 that reported on the effectiveness of online programs of behavioral family intervention. We assessed these findings in light of current research about the relational basis of child development, and the role of parent emotion regulation in the development of child emotion regulatory capacity. The review emphasized the central importance of parent–child relationships in facilitating change in child functioning, and parents’ capacity to regulate emotion as key to optimal child development. The review also indicated that outcomes may be moderated by factors such as methodological, therapeutic, and programmatic characteristics. The review emphasized that parent–child relational development and parental regulation are central in influencing behavioral family systems, and, therefore, child development. These factors therefore warrant further research attention and consideration in the future development of online programs of behavioral family intervention.

## Data Availability

As this study represents a systematic review and meta-analysis of existing research, no new datasets were created and all data generated or analyzed during the study are included in this manuscript.
